# Hormonal replacement therapy in postmenopausal women ≥50 years is associated with the incidence of glaucoma: Evidence from 6576 females in Finland

**DOI:** 10.1111/aos.70138

**Published:** 2026-04-12

**Authors:** Sirpa Loukovaara, Jari Haukka

**Affiliations:** ^1^ Unit of Vitreoretinal Surgery, Department of Ophthalmology Helsinki University Hospital Helsinki Finland; ^2^ Individualized Drug Therapy Research Program University of Helsinki Helsinki Finland; ^3^ Department of Public Health University of Helsinki Helsinki Finland

**Keywords:** glaucoma, glaucoma surgery, hormone replacement therapy, nested case–control, oestrogen, progesterone

## Abstract

**Purpose:**

Hormonal replacement therapy (HRT) in postmenopausal women appears to affect glaucoma risk, but findings are inconsistent. We aimed to explore whether the use of postmenopausal HRT plays a role in glaucoma development in Finland.

**Methods:**

The main outcome variable was glaucoma incidence measured by initiation of special reimbursement due to glaucoma. The population‐based historic cohort consisted of female participants aged ≥50 years whose incident glaucoma was recorded between January 1st, 2015, and December 31st, 2017. The main exposure of interest was postmenopausal HRT with Anatomic Therapeutic Chemical codes G03C (oestrogens), G03D (progestogens), G03F (progestogens and oestrogens in combination). The HRT treatment was initiated during 2001–2010.

Frequencies and odds ratios (ORs) with 95% confidence intervals (CIs) were analyzed with logistic regression models, unadjusted and adjusted for diabetes mellitus, hospital district, socioeconomic status and statin exposure.

**Results:**

Our nested‐case‐control study involved 1096 cases and 5480 age‐matched controls. After adjustment for confounding factors, use of any HRT was associated with an increased risk for the development of glaucoma. The risk was found in estrogen users (adjusted OR, 1.33 [95% CI, 1.17–1.52]), in progestogen users (adjusted OR, 1.25 [95% CI, 1.02–1.53]) and in combinatory estrogen–progesterone users (adjusted OR, 1.19 [95% CI, 1.01–1.39]). In the POAG subgroup, the risk remained in estrogens‐only users (adjusted OR, 1.31 [95% CI, 1.10–1.56]).

**Conclusions:**

Our data suggests that use of any postmenopausal HRT may increase the glaucoma risk, but in POAG subgroup estrogen‐only therapy remained significant. Further research is needed to confirm the associations.

## 1 INTRODUCTION

Glaucoma is among the leading causes of irreversible blindness and a major burden on visual health in the ageing population. Glaucoma is a neurodegenerative disorder of the optic nerve marked by the loss of retinal ganglion cells and axonal degeneration, resulting in irreversible vision impairment. In addition to neurodegeneration, the pathophysiology of glaucoma consists of neuroinflammation and vasculopathy (Mursch‐Edlmayr et al., 2021; Si et al., 2025; Vernazza et al., 2020). Also, genetics, systemic diseases, medications, behavioral and environmental factors appear to contribute to its development.

The prevalence of glaucoma is higher in women than in men. Globally, women comprise approximately 59%–60% of all glaucoma cases. Notably, the primary risk factors of glaucoma for both genders remain advanced age, elevated intraocular pressure and genetics. But gender per se is considered a risk factor for developing glaucoma (Douglass et al., 2023). Exogenous (HRT) for peri‐ and postmenopausal symptoms has been hypothesized to be linked with the gender difference in glaucoma development (Vajaranant et al., 2010). Several studies and a meta‐analysis have indicated that particularly estrogen and estrogen‐only HRT may lower intraocular pressure and offer a neuroprotective effect against glaucoma development (Dewundara et al., 2016; Fotesko et al., 2022; Newman‐Casey et al., 2014; Youngblood et al., 2023). However, despite decades of research, literature remains still inconsistent and controversial (Rudnicka et al., 2006; Zetterberg, 2016; Zhao et al., 2025; Vajaranant et al., 2010).

Although, current evidence seems to point towards a potentially beneficial or neutral effect of HRT on ocular health (Fotesko et al., 2022; Hogan et al., 2024), it is notable, that female sex hormones have also been shown to exacerbate various neurodegenerative diseases, not only retinal neurodegeneration (Kazama et al., 2019). Recently, female sex hormones were shown to be risk factors for worsened outcomes in a model of retinal degeneration (Rowe et al., 2025). Regarding brain and retinal neurodegeneration and neuroinflammation, women may experience worsened outcomes compared to men, but the reasons for this remain poorly understood (Rowe et al., 2025; Zhao et al., 2025).

In our study, we investigated the relationships between exogenous estrogen‐only, progesterone‐only and combinatory oestrogen–progesterone HRT types and incidence of any glaucoma and especially the most common glaucoma subtype, primary open angle glaucoma (POAG) development in postmenopausal women ≥50 years, aiming to find potential associations with glaucoma incidence.

## 2 MATERIALS AND METHODS

### 2.1 Study cohort population

Original study population included 398 708 individuals (Niskanen et al., 2020). To form our study cohort, we included all female participants aged ≥50 years without glaucoma (*n* = 140 473) (Figure 1).

**FIGURE 1:**
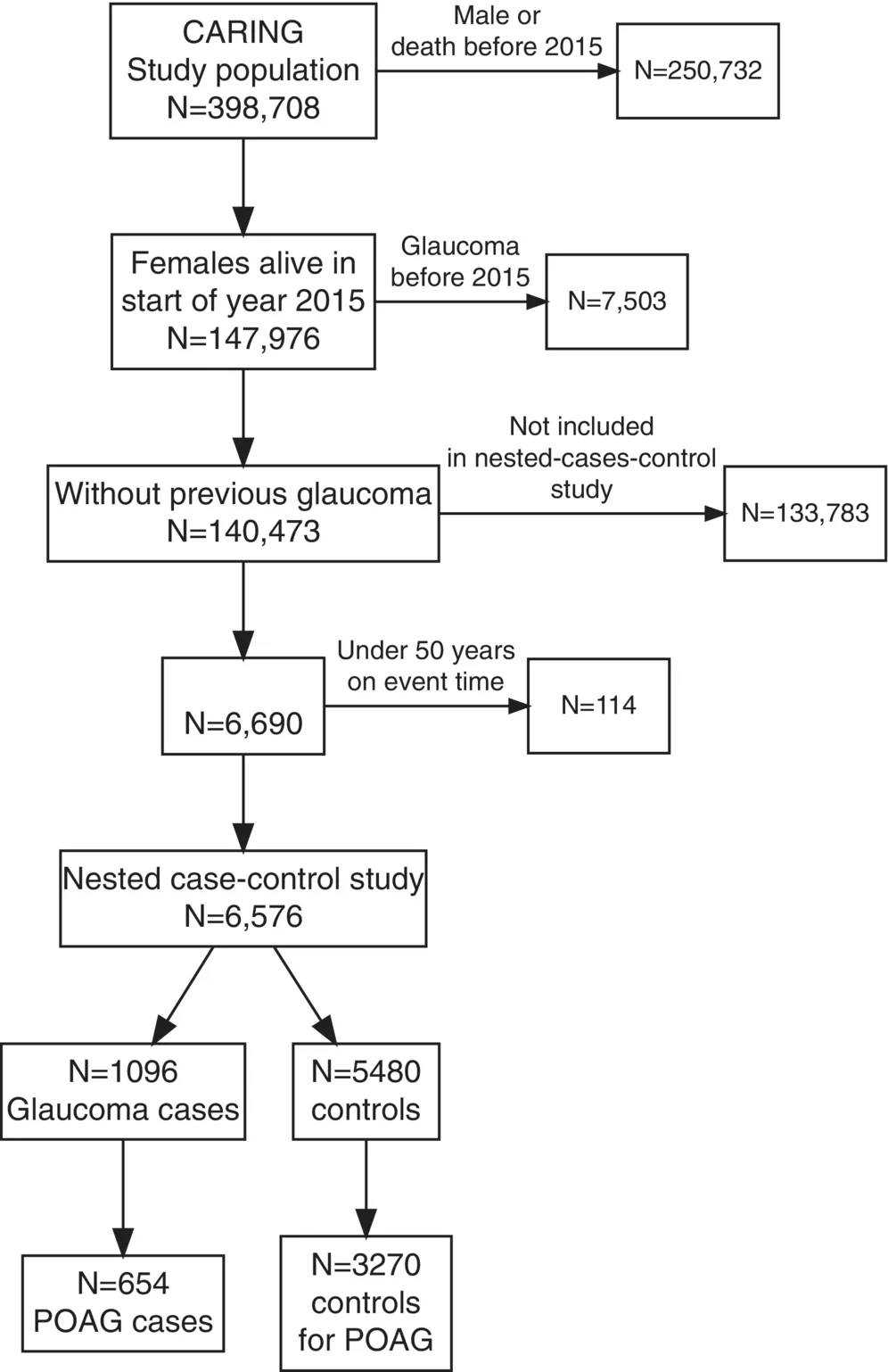
Study population flow chart. Primary open angle glaucoma (POAG) cases (*n* = 654) and their age‐matched controls (*n* = 3270) were analysed as a subgroup.

### 2.2 Exclusion of cases

Exclusion criteria included male sex, death before year 2015 and glaucoma special reimbursement or glaucoma surgery (*n* = 7503) before the year 2015. Glaucoma surgeries were identified from the Finnish Institute for Health and Welfare, Hilmo‐register, with the Nordic Medico‐Statistical Committee (NOMESCO) codes. Herein, we applied the following exclusion criteria for glaucoma surgery (non‐penetrative glaucoma filtration surgery, e.g., deep sclerectomy (DS) procedure codes (CHD60; CHD65)), trabeculectomy (TRE)‐procedure codes (CHD10; CHD15) and glaucoma drainage device (GDD) implantation (CHD50) before 2015.

### 2.3 Nested case–control study design, glaucoma incidence and main exposure

Finally, using the study population of 140 473 female participants without glaucoma, we constructed a nested case–control design (NCC) with cases aged ≥50 years on event, that is, incident glaucoma. Cases were recorded between January 1st, 2015, and December 31st, 2017. Ultimately, our NCC study population consisted of 1096 glaucoma cases and their 5480 age‐matched non‐glaucoma controls (five for each case) (Figure 1). Controls were matched by year of birth with calibre of 1 year, and the NCC incident density sampling was used. Seven cases were identified by glaucoma procedure, and other cases by the glaucoma reimbursement right.

The main outcome variable was the incidence of any glaucoma that was measured by initiation of special reimbursement (i.e. drug prescriptions) due to glaucoma. In a subgroup analysis, other glaucoma subtypes were excluded, and only the International Classification of Diseases (ICD‐10) code H40.1 for primary open angle glaucoma (POAG) was analysed.

The main exposure of interest was postmenopausal HRT. The HRT substances with Anatomic Therapeutic Chemical (ATC) codes were G03C (oestrogens), G03D (progestogens), G03F (progestogens and oestrogens in combination). And in more detail, the ATC codes were as follows: G03CA03 (estradiol), G03CA04 (estriol), G03CX01 (tibolone), G03DA02 (medroxyprogesterone), G03DA04 (progesterone), G03DB01 (dydrogesterone), G03FA01 (norethisterone and oestrogen), G03FA12 (medroxyprogesterone and oestrogen), G03FA14 (dydrogesterone and oestrogen), G03FA17 (drospirenone and oestrogen), G03FB05 (norethisterone and oestrogen), G03FB06 (medroxyprogesterone and oestrogen), G03FB08 (dydrogesterone and oestrogen) or any above mentioned. Follow‐up started on the date of approval of the reimbursement right for HRT medication, documented by the Social Insurance Institute.

We estimated exogenous postmenopausal HRT exposure based on redeemed prescriptions in the past 20 years before the index date (initiation of the special reimbursement date) and the incident glaucoma. HRT exposure was assumed if at least two prescriptions per year were redeemed. We defined the following exposure variables: (A) number of years with HRT exposure (0–20‐year(s)) and (B) indicator for HRT exposure in the last 20 years (0 = no/1 = yes), separately for each ATC code and for any HRT exposure (Tables 2 and 3).

The following potential confounding variables were included in data: diabetes mellitus (no/yes), CARING Study baseline socioeconomic group (*n* = 7), hospital district (*n* = 21) and number of years with statin exposure (ATC C10AA) in the last 20 years (0–20).

### 2.4 Ethics

We obtained approval from the Ethics Committee of the Faculty of Medicine, University of Helsinki, 17 January 2012 (Ref 02/2012). Approvals for data handling were granted by register holders Social Insurance Institute (Kela 29/522/2019), the Institution for Health and Welfare (THL/486/5.05.00/2019) & (THL/3157/14.02.00/2020) and Statistics Finland (TK‐53‐37‐18). All data are pseudonymized and handled under confidentiality agreements. The study adheres to the principles of the Declaration of Helsinki.

### 2.5 Modelling and statistics

We modelled our NCC study with a conditional logistic regression that takes matching into account. Results are presented as frequencies and odds ratios (OR) with 95% confidence intervals (CIs). Statistical analyses were performed by using R (2019).

## 3 RESULTS

In our NCC study, a total of 1096 women developed any glaucoma, and 5480 served as their age‐matched controls (Figure 1). The median age of cases and controls was 75.5 years [69.58, 81.78]. In both groups, the majority of women were pensioners, treated in the Helsinki and Uusimaa district and of Caucasian origin (Table 1). The mean statin exposure was 5.49 (SD 6.09) years among cases and 5.37 (SD 6.01) years among controls.

**TABLE 1:** Basic characteristics of the nested case–control study population.

	Controls	Cases
*N*	5480	1096
Age in years (median [IQR])	75.50 [69.58, 81.58]	75.50 [69.58, 81.58]
G03C (mean (SD))	2.85 (4.61)	3.64 (5.19)
G03D (mean (SD))	0.32 (1.20)	0.41 (1.46)
G03F (mean (SD))	1.15 (2.99)	1.47 (3.59)
G03CA03 (mean (SD))	2.71 (4.55)	3.44 (5.10)
G03CA04 (mean (SD))	0.08 (0.64)	0.09 (0.53)
G03CX01 (mean (SD))	0.05 (0.55)	0.08 (0.70)
G03DA02 (mean (SD))	0.08 (0.57)	0.08 (0.54)
G03DA04 (mean (SD))	0.01 (0.17)	0.01 (0.10)
G03DB01 (mean (SD))	0.05 (0.49)	0.08 (0.82)
G03FA01 (mean (SD))	0.42 (1.70)	0.53 (2.04)
G03FA12 (mean (SD))	0.14 (0.98)	0.20 (1.29)
G03FA14 (mean (SD))	0.04 (0.45)	0.08 (0.78)
G03FA17 (mean (SD))	0.02 (0.42)	0.00 (0.07)
G03FB05 (mean (SD))	0.23 (1.07)	0.27 (1.17)
G03FB06 (mean (SD))	0.23 (1.13)	0.26 (1.20)
G03FB08 (mean (SD))	0.05 (0.44)	0.08 (0.75)
C10AA (mean (SD))	5.37 (6.01)	5.49 (6.09)
DM = yes (*n*, %)	2635 (48.1)	500 (45.6)
Socioeconomic group (*n*, %)		
Upper‐level employees	323 (5.9)	69 (6.3)
Self‐employed	219 (4.0)	37 (3.4)
Lower‐level employees	824 (15.0)	188 (17.2)
Manual workers	498 (9.1)	99 (9.0)
Students	19 (0.3)	5 (0.5)
Pensioners	3149 (57.5)	622 (56.8)
Others	448 (8.2)	76 (6.9)
Hospital district (*n*, %)		
Helsinki and Uusimaa	1223 (22.3)	277 (25.3)
Varsinais‐Suomi	447 (8.2)	75 (6.8)
Satakunta	237 (4.3)	44 (4.0)
Kanta‐Häme	171 (3.1)	35 (3.2)
Pirkanmaa	506 (9.2)	110 (10.0)
Päijät‐Häme	237 (4.3)	39 (3.6)
Kymenlaakso	223 (4.1)	54 (4.9)
Etelä‐Karjala	147 (2.7)	31 (2.8)
Etelä‐Savo	137 (2.5)	25 (2.3)
Itä‐Savo	69 (1.3)	12 (1.1)
Pohjois‐Karjala	211 (3.9)	38 (3.5)
Pohjois‐Savo	314 (5.7)	52 (4.7)
Keski‐Suomi	270 (4.9)	85 (7.8)
Etelä‐Pohjanmaa	265 (4.8)	38 (3.5)
Vaasa	165 (3.0)	36 (3.3)
Keski‐Pohjanmaa	90 (1.6)	13 (1.2)
Pohjois‐Pohjanmaa	357 (6.5)	62 (5.7)
Kainuu	119 (2.2)	30 (2.7)
Länsi‐Pohja	109 (2.0)	13 (1.2)
Lappi	157 (2.9)	23 (2.1)
Ahvenanmaa	26 (0.5)	4 (0.4)
Indicator of any HRT (%)	2912 (53.1)	664 (60.6)
Indicator of G03C (%)	2452 (44.7)	568 (51.8)
Indicator of G03D (%)	584 (10.7)	143 (13.0)
Indicator of G03F (%)	1096 (20.0)	253 (23.1)
Indicator of G03CA03 (%)	2304 (42.0)	532 (48.5)
Indicator of G03CA04 (%)	195 (3.6)	51 (4.7)
Indicator of G03CX01 (%)	62 (1.1)	18 (1.6)
Indicator of G03DA02 (%)	165 (3.0)	35 (3.2)
Indicator of G03DA04 (%)	19 (0.3)	8 (0.7)
Indicator of G03DB01 (%)	102 (1.9)	26 (2.4)
Indicator of G03FA01 (%)	541 (9.9)	121 (11.0)
Indicator of G03FA12 (%)	176 (3.2)	40 (3.6)
Indicator of G03FA14 (%)	71 (1.3)	21 (1.9)
Indicator of G03FA17 (%)	28 (0.5)	3 (0.3)
Indicator of G03FB05 (%)	357 (6.5)	88 (8.0)
Indicator of G03FB06 (%)	342 (6.2)	78 (7.1)
Indicator of G03FB08 (%)	92 (1.7)	23 (2.1)

*Note*: The HRT substances with Anatomic Therapeutic Chemical (ATC) codes were G03C (oestrogens), G03D (progestogens), G03F (progestogens and oestrogens in combination). And in more detail the ATC codes were as follows: G03CA03 (estradiol), G03CA04 (estriol), G03CX01 (tibolone), G03DA02 (medroxyprogesterone), G03DA04 (progesterone), G03DB01 (dydrogesterone), G03FA01 (norethisterone and oestrogen), G03FA12 (medroxyprogesterone and oestrogen), G03FA14 (dydrogesterone and oestrogen), G03FA17 (drospirenone and oestrogen), G03FB05 (norethisterone and oestrogen), G03FB06 (medroxyprogesterone and oestrogen), G03FB08 (dydrogesterone and oestrogen).

All in all, among the incident glaucoma group, 60.6% of women used any HRT within the 20‐year study period compared to 53.1% among controls. As regards HRT use, oestrogens‐only were used among 568 (51.8%) cases and 2452 (44.7%) controls (Table 1). The mean exposure time of oestrogen use was 3.64 (SD 5.19) years among cases and 2.85 (SD 4.61) years among controls.

After adjustment for confounding factors, use of any HRT was associated with an increased risk for the development of glaucoma. Furthermore, the increased risk was found in oestrogen‐only users (unadjusted OR, 1.34 [95% CI, 1.18–1.53] and adjusted OR, 1.33 [95% CI, 1.17–1.52]), in progestogen‐users (unadjusted OR, 1.27 [95% CI, 1.04–1.55] and adjusted OR, 1.25 [95% CI, 1.02–1.53]) and in combinatory progestogen–oestrogen users (unadjusted OR, 1.22 [95% CI, 1.04–1.43] and adjusted OR, 1.19 [95% CI, 1.01–1.39]) (Table 2). The main findings remained when analysed according to exogenous HRT exposure per 1 year (0–20) (Table 3).

**TABLE 2:** Frequencies and odds ratios (OR) with 95% CI for the indicator of exogenous HRT exposure in the glaucoma cohort (*N* = 1096) and their age‐matched controls (*N* = 5480).

	Control	Cases	Unadjusted	Adjusted
Indicator of any HRT				
No	2568	432	(reference)	(reference)
Yes	2912	664	1.38 (1.20–1.57)	1.36 (1.19–1.56)
Indicator of G03C				
No	3028	528	(reference)	(reference)
Yes	2452	568	1.34 (1.18–1.53)	1.33 (1.17–1.52)
Indicator of G03D				
No	4896	953	(reference)	(reference)
Yes	584	143	1.27 (1.04–1.55)	1.25 (1.02–1.53)
Indicator of G03F				
No	4384	843	(reference)	(reference)
Yes	1096	253	1.22 (1.04–1.43)	1.19 (1.01–1.39)
Indicator of G03CA03				
No	3176	564	(reference)	(reference)
Yes	2304	532	1.32 (1.15–1.50)	1.30 (1.14–1.49)
Indicator of G03CA04				
No	5285	1045	(reference)	(reference)
Yes	195	51	1.33 (0.97–1.82)	1.35 (0.98–1.85)
Indicator of G03CX01				
No	5418	1078	(reference)	(reference)
Yes	62	18	1.46 (0.86–2.48)	1.39 (0.82–2.37)
Indicator of G03DA02				
No	5315	1061	(reference)	(reference)
Yes	165	35	1.06 (0.73–1.54)	1.04 (0.72–1.51)
Indicator of G03DA04				
No	5461	1088	(reference)	(reference)
Yes	19	8	2.14 (0.93–4.93)	2.09 (0.90–4.85)
Indicator of G03DB01				
No	5378	1070	(reference)	(reference)
Yes	102	26	1.28 (0.83–1.98)	1.22 (0.79–1.90)
Indicator of G03FA01				
No	4939	975	(reference)	(reference)
Yes	541	121	1.13 (0.92–1.40)	1.11 (0.90–1.38)
Indicator of G03FA12				
No	5304	1056	(reference)	(reference)
Yes	176	40	1.14 (0.80–1.63)	1.11 (0.78–1.58)
Indicator of G03FA14				
No	5409	1075	(reference)	(reference)
Yes	71	21	1.49 (0.91–2.45)	1.45 (0.88–2.39)
Indicator of G03FA17				
No	5452	1093	(reference)	(reference)
Yes	28	3	0.53 (0.16–1.76)	0.51 (0.15–1.69)
Indicator of G03FB05				
No	5123	1008	(reference)	(reference)
Yes	357	88	1.27 (0.99–1.62)	1.23 (0.95–1.57)
Indicator of G03FB06				
No	5138	1018	(reference)	(reference)
Yes	342	78	1.16 (0.89–1.50)	1.11 (0.86–1.45)
Indicator of G03FB08				
No	5388	1073	(reference)	(reference)
Yes	92	23	1.28 (0.79–2.08)	1.25 (0.77–2.04)

*Note*: Unadjusted and adjusted OR for diabetes mellitus, socioeconomic group, hospital district and statin exposure calculated with conditional logistic regression. The HRT substances with Anatomic Therapeutic Chemical (ATC) codes were G03C (oestrogens), G03D (progestogens), G03F (progestogens and oestrogens in combination). And in more detail the ATC codes were as follows: G03CA03 (estradiol), G03CA04 (estriol), G03CX01 (tibolone), G03DA02 (medroxyprogesterone), G03DA04 (progesterone), G03DB01 (dydrogesterone), G03FA01 (norethisterone and oestrogen), G03FA12 (medroxyprogesterone and oestrogen), G03FA14 (dydrogesterone and oestrogen), G03FA17 (drospirenone and oestrogen), G03FB05 (norethisterone and oestrogen), G03FB06 (medroxyprogesterone and oestrogen), G03FB08 (dydrogesterone and oestrogen).

**TABLE 3:** Odds ratios (OR) with 95% CI for exogenous HRT exposure per one year (0–20) in the glaucoma cohort (N = 1096) and their age‐matched controls (N = 5480).

ATC code	Unadjusted	Adjusted
G03C	1.03 (1.02–1.05)	1.03 (1.02–1.05)
G03D	1.06 (1.01–1.11)	1.05 (1.00–1.10)
G03F	1.03 (1.01–1.05)	1.03 (1.01–1.05)
G03CA03	1.03 (1.02–1.05)	1.03 (1.02–1.05)
G03CA04	1.01 (0.92–1.12)	1.02 (0.92–1.13)
G03CX01	1.08 (0.98–1.19)	1.07 (0.97–1.18)
G03DA02	1.00 (0.89–1.12)	0.99 (0.89–1.11)
G03DA04	1.05 (0.73–1.53)	1.04 (0.72–1.51)
G03DB01	1.08 (0.98–1.19)	1.08 (0.98–1.19)
G03FA01	1.04 (1.00–1.07)	1.03 (1.00–1.07)
G03FA12	1.05 (1.00–1.11)	1.05 (0.99–1.10)
G03FA14	1.13 (1.03–1.25)	1.13 (1.02–1.25)
G03FA17	0.71 (0.41–1.22)	0.70 (0.40–1.23)
G03FB05	1.04 (0.98–1.10)	1.03 (0.97–1.09)
G03FB06	1.02 (0.97–1.08)	1.02 (0.96–1.07)
G03FB08	1.13 (1.01–1.26)	1.12 (1.00–1.25)

*Note*: Unadjusted and adjusted OR for diabetes mellitus, socioeconomic group, hospital district and statin exposure calculated with conditional logistic regression. The HRT substances with Anatomic Therapeutic Chemical (ATC) codes were G03C (oestrogens), G03D (progestogens), G03F (progestogens and oestrogens in combination). And in more detail the ATC codes were as follows: G03CA03 (estradiol), G03CA04 (estriol), G03CX01 (tibolone), G03DA02 (medroxyprogesterone), G03DA04 (progesterone), G03DB01 (dydrogesterone), G03FA01 (norethisterone and oestrogen), G03FA12 (medroxyprogesterone and oestrogen), G03FA14 (dydrogesterone and oestrogen), G03FA17 (drospirenone and oestrogen), G03FB05 (norethisterone and oestrogen), G03FB06 (medroxyprogesterone and oestrogen), G03FB08 (dydrogesterone and oestrogen).

### 3.1 POAG subgroup

Altogether, 654 new cases of POAG were diagnosed during the study period. For NCC design, 3270 women served as their age‐matched controls (Figure 1). Also, in subgroup analysis, use of any HRT was found to be associated with an increased risk for development of POAG (unadjusted OR, 1.39 [95% CI, 1.16–1.66] and adjusted OR, 1.35 [95% CI, 1.13–1.62]) (Table 4). But in more detailed analysis, the risk remained higher in oestrogen‐only users (unadjusted OR, 1.35 [95% CI, 1.13–1.60] and adjusted OR, 1.31 [95% CI, 1.10–1.56]). Especially, estradiol use was associated with increased risk of POAG.

**TABLE 4:** Frequencies and odds ratios (OR) with 95% CI for the indicator of HRT exposure in primary open angle glaucoma (POAG) subgroup and their age‐matched controls.

	Control	Cases	Unadjusted	Adjusted
Indicator of any HRT				
No	1488	248	(reference)	(reference)
Yes	1782	406	1.39 (1.16–1.66)	1.35 (1.13–1.62)
Indicator of G03C				
No	1767	307	(reference)	(reference)
Yes	1503	347	1.35 (1.13–1.60)	1.31 (1.10–1.56)
Indicator of G03D				
No	2906	565	(reference)	(reference)
Yes	364	89	1.27 (0.98–1.64)	1.25 (0.97–1.62)
Indicator of G03F				
No	2603	503	(reference)	(reference)
Yes	667	151	1.18 (0.96–1.45)	1.16 (0.94–1.43)
Indicator of G03CA03				
No	1845	331	(reference)	(reference)
Yes	1425	323	1.28 (1.08–1.52)	1.23 (1.03–1.47)
Indicator of G03CA04				
No	3161	623	(reference)	(reference)
Yes	109	31	1.45 (0.96–2.20)	1.53 (1.00–2.33)
Indicator of G03CX01				
No	3232	643	(reference)	(reference)
Yes	38	11	1.46 (0.74–2.87)	1.39 (0.70–2.76)
Indicator of G03DA02				
No	3171	630	(reference)	(reference)
Yes	99	24	1.22 (0.78–1.91)	1.20 (0.76–1.89)
Indicator of G03DA04				
No	3259	650	(reference)	(reference)
Yes	11	4	1.86 (0.58–5.97)	1.71 (0.52–5.61)
Indicator of G03DB01				
No	3205	635	(reference)	(reference)
Yes	65	19	1.47 (0.88–2.47)	1.47 (0.87–2.48)
Indicator of G03FA01				
No	2936	575	(reference)	(reference)
Yes	334	79	1.21 (0.93–1.57)	1.21 (0.93–1.58)
Indicator of G03FA12				
No	3163	627	(reference)	(reference)
Yes	107	27	1.28 (0.83–1.97)	1.22 (0.79–1.90)
Indicator of G03FA14				
No	3227	638	(reference)	(reference)
Yes	43	16	1.90 (1.06–3.42)	1.92 (1.06–3.49)
Indicator of G03FA17				
No	3253	653	(reference)	(reference)
Yes	17	1	0.29 (0.04–2.21)	0.27 (0.04–2.04)
Indicator of G03FB05				
No	3052	603	(reference)	(reference)
Yes	218	51	1.19 (0.86–1.65)	1.16 (0.84–1.61)
Indicator of G03FB06				
No	3083	605	(reference)	(reference)
Yes	187	49	1.34 (0.97–1.87)	1.29 (0.92–1.80)
Indicator of G03FB08				
No	3212	641	(reference)	(reference)
Yes	58	13	1.14 (0.60–2.14)	1.07 (0.56–2.04)

*Note*: Unadjusted and adjusted OR for diabetes mellitus, socioeconomic group, hospital district and statin exposure calculated with conditional logistic regression. The HRT substances with Anatomic Therapeutic Chemical (ATC) codes were G03C (oestrogens), G03D (progestogens), G03F (progestogens and oestrogens in combination). And in more detail the ATC codes were as follows: G03CA03 (estradiol), G03CA04 (estriol), G03CX01 (tibolone), G03DA02 (medroxyprogesterone), G03DA04 (progesterone), G03DB01 (dydrogesterone), G03FA01 (norethisterone and oestrogen), G03FA12 (medroxyprogesterone and oestrogen), G03FA14 (dydrogesterone and oestrogen), G03FA17 (drospirenone and oestrogen), G03FB05 (norethisterone and oestrogen), G03FB06 (medroxyprogesterone and oestrogen), G03FB08 (dydrogesterone and oestrogen).

But no significant associations were found with progestogen or combined oestrogen‐and‐progesterone HRT groups.

However, in a specific dydrogesterone–oestrogen treated subgroup, there was a significant risk association with POAG development (unadjusted OR, 1.90 [95% CI, 1.06–3.42] and adjusted OR, 1.92 [95% CI, 1.06–3.49]).

Finally, the main findings remained in the POAG subgroup treated with oestrogen‐only and estradiol when analysed according to exogenous HRT exposure per 1 year (0–20) (Table 5).

**TABLE 5:** Odds ratios (OR) with 95% CI for HRT exposure per one year (0–20) in the primary open angle glaucoma (POAG) subgroup and their age‐matched controls.

ATC code	Unadjusted	Adjusted
G03C	1.03 (1.01–1.05)	1.03 (1.01–1.04)
G03D	1.05 (0.99–1.12)	1.05 (0.99–1.11)
G03F	1.04 (1.01–1.07)	1.04 (1.01–1.06)
G03CA03	1.03 (1.01–1.05)	1.02 (1.01–1.04)
G03CA04	1.04 (0.91–1.20)	1.07 (0.92–1.23)
G03CX01	1.08 (0.95–1.24)	1.07 (0.93–1.23)
G03DA02	1.02 (0.88–1.18)	1.01 (0.87–1.17)
G03DA04	1.05 (0.66–1.68)	1.01 (0.64–1.62)
G03DB01	1.10 (0.99–1.22)	1.09 (0.99–1.21)
G03FA01	1.05 (1.01–1.09)	1.05 (1.01–1.09)
G03FA12	1.06 (0.99–1.13)	1.05 (0.98–1.12)
G03FA14	1.16 (1.03–1.29)	1.16 (1.04–1.30)
G03FA17	0.53 (0.14–1.98)	0.51 (0.13–2.06)
G03FB05	1.03 (0.96–1.11)	1.03 (0.95–1.11)
G03FB06	1.05 (0.98–1.13)	1.04 (0.97–1.12)
G03FB08	1.14 (0.98–1.32)	1.13 (0.97–1.32)

*Note*: Unadjusted and adjusted OR for diabetes mellitus, socioeconomic group, hospital district and statin exposure calculated with conditional logistic regression. The HRT substances with Anatomic Therapeutic Chemical (ATC) codes were G03C (oestrogens), G03D (progestogens), G03F (progestogens and oestrogens in combination). And in more detail the ATC codes were as follows: G03CA03 (estradiol), G03CA04 (estriol), G03CX01 (tibolone), G03DA02 (medroxyprogesterone), G03DA04 (progesterone), G03DB01 (dydrogesterone), G03FA01 (norethisterone and oestrogen), G03FA12 (medroxyprogesterone and oestrogen), G03FA14 (dydrogesterone and oestrogen), G03FA17 (drospirenone and oestrogen), G03FB05 (norethisterone and oestrogen), G03FB06 (medroxyprogesterone and oestrogen), G03FB08 (dydrogesterone and oestrogen).

### 3.2 Adjusted splines

Next, we defined and fitted the splines to the data and visualized the splines with graphs to better understand the effect of each used HRT, divided according to ACT codes, on glaucoma development during the study period (Figure S1). This analysis using splines showed that longer exposure to HRT– especially with oestrogens‐only therapy (G03C)– was associated with a higher risk of glaucoma.

Finally, we defined and fitted the spines presenting any HRT exposure (Figure S2). Horizontal axis zero shows no exposure at all and demonstrates how higher HRT exposure is consistently associated with a higher risk of glaucoma.

## 4 DISCUSSION

The relationship between postmenopausal HRT and the risk of glaucoma in women over 50 has been explored in several studies, with a focus on the potential neuroprotective effects of oestrogen‐only against glaucoma (Fotesko et al., 2022). Contradicting previous studies, our findings suggest that three types of exogenous postmenopausal HRT (oestrogen‐only, progestogen‐only and combinatory oestrogen–progestogen) are associated with the increased incidence of any glaucoma in a Finnish population‐based cohort. However, in the sub‐analysis of POAG women only, our findings suggest that merely exogenous oestrogen‐only therapy may affect the likelihood of developing POAG. And in more detailed analysis, estradiol and dydrogesterone–oestrogen therapies seemed to be associated with an increased risk of POAG.

In addition to glaucoma development, gender/sex has been implicated as a modifying factor in the pathogenesis of neurodegenerative brain diseases, such as Alzheimer's disease, Parkinson's disease, as well as eye disorders, age‐related macular degeneration and diabetic retinopathy (Chen et al., 2024; Kazama et al., 2019; Lopez‐Lee et al., 2024; Nuzzi et al., 2018). During aging, women undergo distinct processes, marked by different hormonal changes, cellular activities and age‐associated health conditions compared with males. Understanding better the mechanism of neurodegeneration in glaucoma among females may help to develop novel therapeutic interventions. Today, emerging studies have also suggested a significant role for the gut–brain axis in glaucoma development (Kammoun et al., 2024).

Menopause is a major biological life event that affects all women. It coincides with a decrease in circulating sex hormones, such as oestrogen. During perimenopause, the oestrogen levels become highly variable by initiating oestrogen‐related dysfunction in metabolic, inflammatory and sensory processing‐related molecular pathways (McCarthy & Raval, 2020). Since menopausal transition is an inflammatory event, it can be associated with neuroinflammation in glaucoma (Quaranta et al., 2021). Although some studies have suggested that statins may slow the onset of OAG, long‐term statin exposure did not have any beneficial association in our study.

Commonly used HRT drug combinations are oestrogen combined with dydrogesterone (synthetic progesterone). Our study identified an increased risk of POAG in Finnish women with HRT‐containing combined oestrogen/synthetic progesterone, suggesting that adverse effects of HRT could also be linked to the specific hormones used for postmenopausal therapy. Thus, our results seem to contradict the recent systematic review findings, where no significant associations were found with combined oestrogen–progesterone HRT use and OAG development (Madjedi et al., 2022).

In epidemiological studies, there are specific limitations in identifying glaucoma incidence rates from administrative medication data. However, the Finnish nationwide medical insurance register provides a solid source for identifying beneficiaries with glaucoma, basing glaucoma diagnoses on drug prescriptions. Since a proportion of IOP‐reducing drugs are known to be prescribed to people without definitive glaucoma, it is possible that there could also be female patients with ocular hypertension without definitive glaucoma in our study.

Although the association between HRT and glaucoma observed in our study is of interest, several aspects of the study raise questions about the strength of our conclusions. It may be that women on HRT might have more frequent medical visits, increasing the likelihood of POAG detection compared with non‐HRT‐users. Also, the small sample sizes could lead to unstable estimates, exaggerating effect sizes and confidence intervals. In addition, HRT use might be correlated with other health behaviours or conditions that themselves affect glaucoma risk in addition to diabetes, socioeconomic background, hospital district and number of years with statin therapy (e.g., hypertension, BMI, smoking).

To our knowledge, this is the largest study to assess a potential relationship between postmenopausal HRT use and glaucoma development in Finland. Our population‐based study covered all geographic regions, that is, 21 hospital districts in Finland. Other strengths of our study included nested case–control design, knowledge on 20‐year exposure of all specific types of available HRT options and all socioeconomic groups. In the future, further research is needed to confirm the associations and to better understand the complex relationship between HRT use and glaucoma incidence.

## AUTHOR CONTRIBUTIONS

Jari Haukka, PhD, had full access to all the data in the study and takes responsibility for the integrity of the data and the accuracy of the data analysis.

## Supporting information


Figure S1.

Figure S2.
