# Supplementary material for: Hormonal replacement therapy in postmenopausal women ≥50 years is associated with the incidence of glaucoma: Evidence from 6576 females in Finland
Source: Acta Ophthalmol. 2026 Apr 12;104(7):742–9. doi: 10.1111/aos.70138 (PMC13651931; doi:10.1111/aos.70138)
Supplement: Supplementary file 1 [file AOS-104-742-s001.pdf]

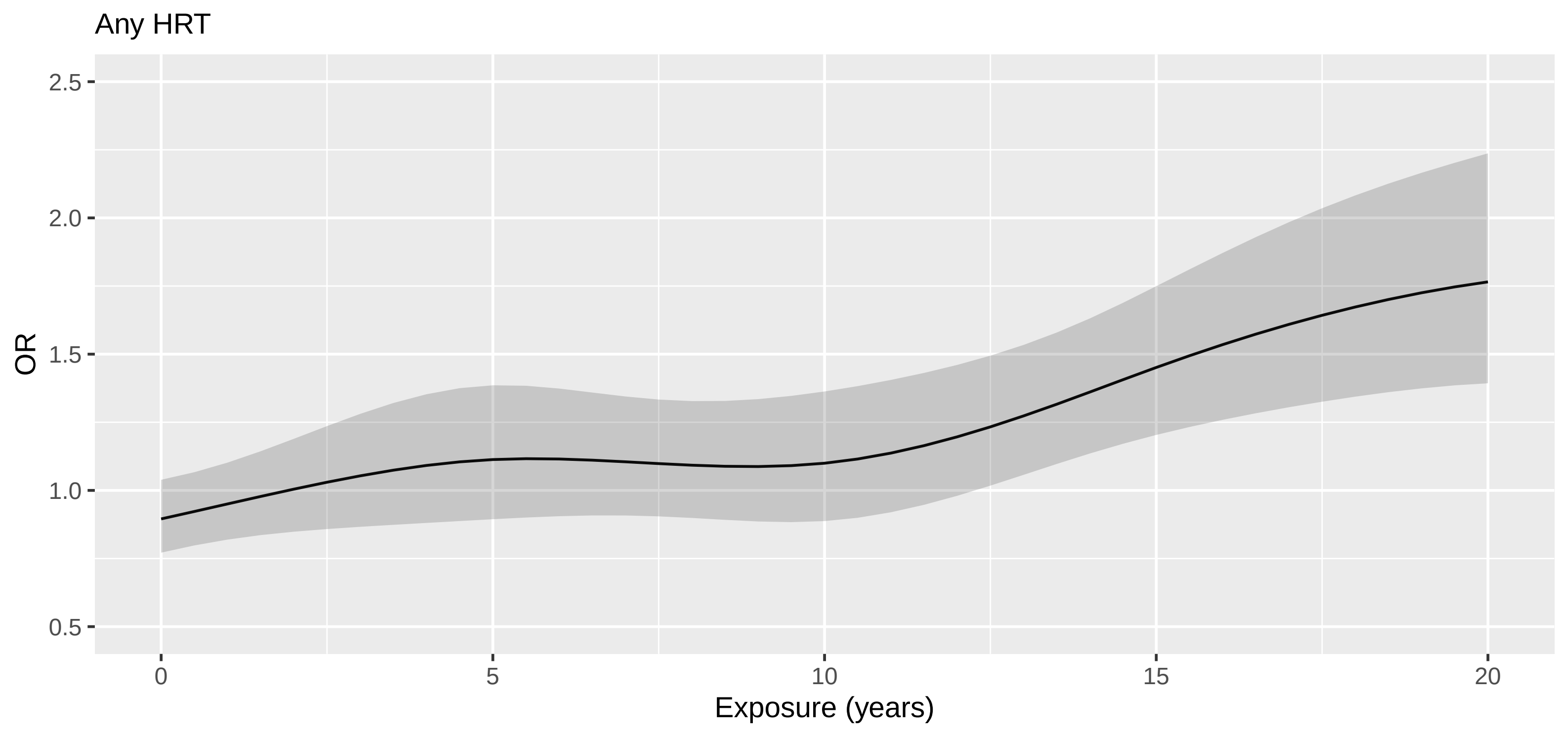

Any HRT
OR
2.5
2.0
1.5
1.0
0.5
0
5
10
15
20
Exposure (years)
