# Supplementary material for: Hormonal replacement therapy in postmenopausal women ≥50 years is associated with the incidence of glaucoma: Evidence from 6576 females in Finland
Source: Acta Ophthalmol. 2026 Apr 12;104(7):742–9. doi: 10.1111/aos.70138 (PMC13651931; doi:10.1111/aos.70138)
Supplement: Supplementary file 2 [file AOS-104-742-s002.pdf]

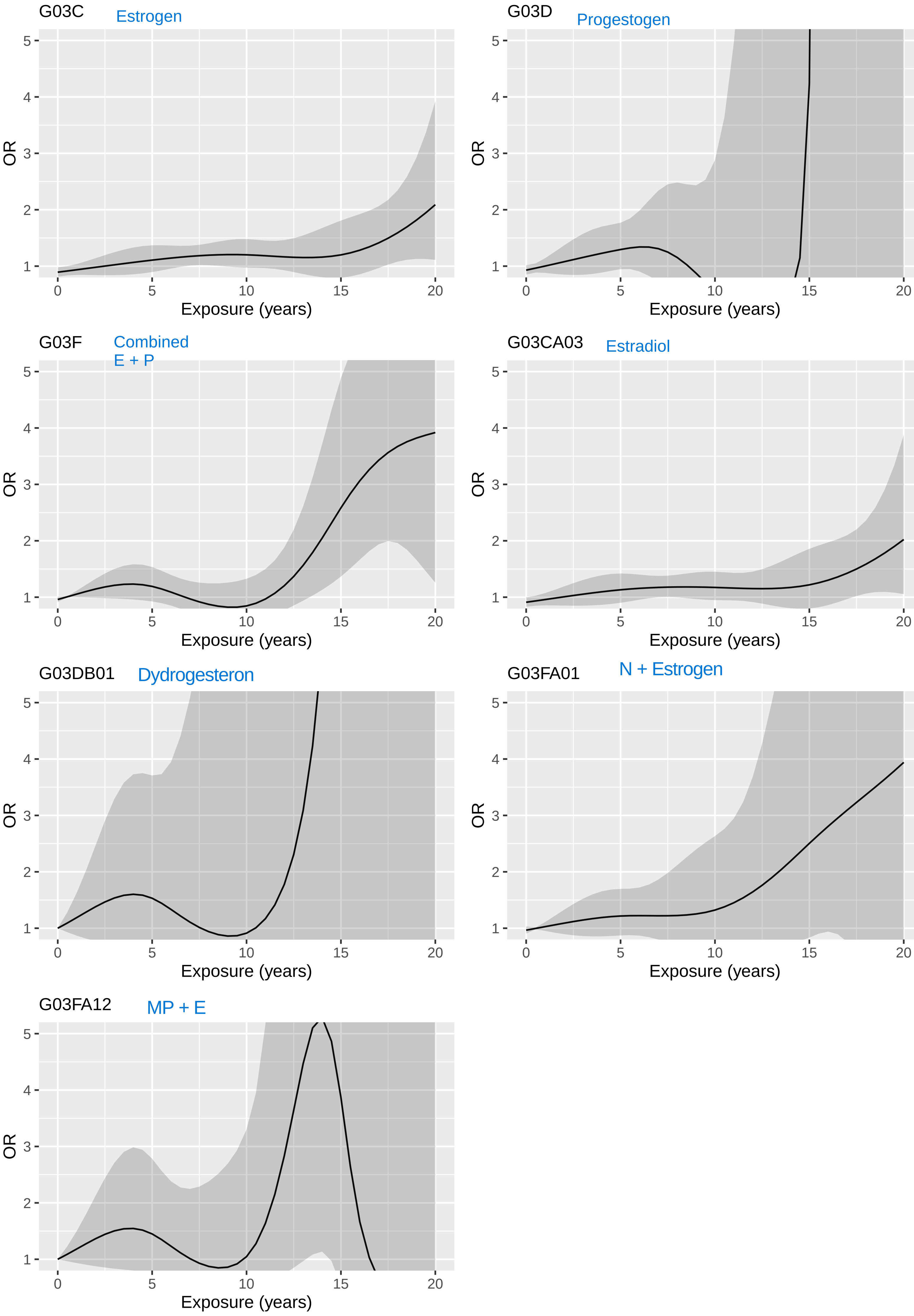

G03C
Estrogen
G03D
Progestogen
G03F
Combined
E + P
G03CA03
Estradiol
G03DB01
Dydrogesteron
G03FA01
N + Estrogen
G03FA12
MP + E
OR
Exposure (years)
1
2
3
4
5
0
5
10
15
20
